# An Exploration on the Suitability of Airborne Carbonyl Compounds Analysis in relation to Differences in Instrumentation (GC-MS versus HPLC-UV) and Standard Phases (Gas versus Liquid)

**DOI:** 10.1155/2014/308405

**Published:** 2014-02-25

**Authors:** Ki-Hyun Kim, Jan E. Szulejko, Yong-Hyun Kim, Min-Hee Lee

**Affiliations:** Department of Civil and Environmental Engineering, Hanyang University, 222 Wangsimni-Ro, Seoul 133-791, Republic of Korea

## Abstract

The relative performance figure of merits was investigated for the two most common analytical methods employed for carbonyl compounds (CC), for example, between high performance liquid chromatography (HPLC)-UV detector (with 2,4-dinitrophenylhydrazine (DNPH) derivatization) and thermal desorption (TD)-gas chromatography (GC)-mass spectrometry (MS) (without derivatization). To this end, the suitability of each method is assessed by computing the relative recovery (RR) between the gas- and liquid-phase standards containing a suite of CC such as formaldehyde (FA), acetaldehyde (AA), propionaldehyde (PA), butyraldehyde (BA), isovaleraldehyde (IA), and valeraldehyde (VA) along with benzene (B) as a recovery reference for the GC method. The results confirm that a TD-GC-MS is advantageous to attain the maximum recovery for the heavier CCs (i.e., with molecular weights (MW) above BA−MW ≥ 74). On the other hand, the HPLC-UV is favorable for the lighter CCs (like FA and AA) with the least bias. Such compound-specific responses for each platform are validated by relative ordering of CCs as a function of response factor (RF), method detection limit (MDL), and recovery pattern. It is thus desirable to understand the advantages and limitations of each method to attain the CC data with the least experimental bias.

## 1. Introduction

The analysis of trace components in a gaseous matrix including ambient air has gained an increasing amount of attention since the 1950's, especially in the field of environmental chemistry, for example, volatile organic compounds (VOC) [1], owing to their potential impacts on human health and to a growing demand for rigorous air quality regulation. Among the wide array of VOC groups, carbonyl compounds (CC) are known to play an important role in secondary organic aerosol (SOA) formation and the associated alteration of climate conditions [2]. Lathière et al. [3] have estimated the total global biogenic volatile organic compound (BVOC) emissions to be 752 Tg C/year (as carbon) for the period 1983–1995; for instance, the contribution of acetone was estimated as 42 Tg C/year. The H-abstraction and NO_2_-addition reactions of aldehydes were studied theoretically to gain insight into the formation of more potent pollutants (e.g., see [4]).

The increasing use of ethanol biofuels such as E85 gasoline is projected to increase acetaldehyde and the associated cancer prevalence in the Los Angeles area in the U.S. [5]. CCs are emitted/produced from cooking activities [6], household furniture [7], biomass fuel combustion [8], sports beverage containers [9], drinking water (as disinfection byproducts (DBP)) [10], and industrial sources [11]. Recently, carbonyl emissions from vehicles running on petroleum or biobased fuels have become a major area in pollution study [12–14] and life-cycle-analysis has been used to determine which fuels (petroleum versus bio) are actually more harmful to the environment, for example, [15, 16]. Ongoing European reforestation projects are projected to increase formaldehyde, acetaldehyde, and acetone biogenic emissions in Europe by 56% (minimally, globally) and potentially cause European regional climate change [3].

To date, a number of analytical approaches have been proposed and employed for the quantitative analysis of trace CC in ambient air. Among those options, the use of the 2,4-dinitrophenylhydrazine (DNPH) cartridge combined with high performance liquid chromatography (HPLC)-UV detection is often considered the most favorable experimental choice [6, 7, 12, 13]. It is however reported that gas chromatographic (GC) analysis combined with pentafluorophenylhydrazine (PFPH) derivatization can yield more reliable data than the HPLC method, when both methods are compared on a parallel basis [17]. Despite such efforts, the efficient detection of CCs in ambient air (and on exhaled breath as potential disease diagnostic biomarkers) still remains a formidable challenge [18].

To facilitate their detection in ambient air, one needs to consider various factors involved in their sampling under field conditions: (a) relative humidity, (b) sampling time, (c) collection (derivatization) efficiency, and (d) O_3_ denuders that oxidize NO to NO_2_. Concerns on the use of DNPH/sorbent cartridges to sample FA, AA, and acrolein in environmental air over extended periods (up to 24 h) have been reviewed with emphasis on O_3_ and NO_2_ reactions with DNPH yielding interfering artifacts in the subsequent analysis and sampling time [19]. For example, the AA/FA collection efficiency (CE) from air samples is near 100% for sampling periods ranging from minutes to a few hours. In contrast, for 24 h sampling, the reported CE was only 1–62% and the lower CE is scientifically unexplainable [19]. In contrast, in case of heavier CCs other than AA/FA, large reductions in collection efficiency were also seen from DNPH cartridge method for a normal sampling duration of a few hours [20]. There are a number of reviews that describe a wide array of experimental options to carry our quantitative analysis of CCs [21–23] and VOCs in general [24, 25]. A 2009 review [26] discusses CC detection methods and their limit of detection (LOD). A diverse range of chromophoric and derivatization reagents has been developed and used to facilitate CC detection by HPLC and GC. The use of hydrazine reagents in environmental analysis has been critically reviewed; DNPH is recognized as an international standard [27].

In an effort to characterize the basic methodological approaches available in the CC analysis, we investigated the experimental compatibilities and differences between HPLC and GC methods. To this end, a series of calibration experiments were conducted by both systems using identical standards containing 5 CCs (acetaldehyde (AA), propionaldehyde (PA), butyraldehyde (BA), isovaleraldehyde (IV), and valeraldehyde (VA)) prepared in both gas and liquid phases. The experimental results were then evaluated with respect to the sensitivities or reproducibilities across different carbonyls. In the course of this comparative study, preconcentration of CCs by each system was treated by their basic tools such as cartridge derivatization (HPLC-UV) and sorbent tube trapping (GC). Based on this comparative study, we explore the fundamental properties of each experimental method for CC and discuss their advantages and disadvantages.

## 2. Materials and Methods

### 2.1. The Significance of Relative Recovery in CC Analysis between Different Standard Phases

The basic information (e.g., molecular formula, molecular weight, density, and chemical structure) of the target carbonyls investigated in this study is briefly summarized in Table 1. To conduct a calibration-based analysis for the target CCs for a parallel comparison, their working standards (WS) prepared in both gas and liquid phase were analyzed by loading comparable quantities of the target analytes. (See Section 2.2 for the details of standard preparation.) The basic operation conditions for each instrumental setup are summarized in Table  1S (see Table S1 in Supplementary Materials available online at http://dx.doi.org/10.1155/2014/308405).

Note that the analysis of gas standards can be made with similar treatment steps for each method such as derivatization via cartridge sampler (HPLC) and collection via sorbent tube (ST) for thermal desorption (GC). Likewise, the GC-based analysis of liquid standard can be made, similar to real samples, by employing the combined application of sorbent tube collection and thermal desorption treatment. In contrast, it is not the case for the HPLC, as the analysis of the liquid standard can be significantly complicated due to the involvement of reactions leading to their derivatization. Hence, the HPLC analysis could be subject to relatively large biases in the quantitative analysis, if evaluated in terms of compatibility between standard and sample.

Considering all these complicated factors involved in the CC analysis, the relative recovery (RR) between different standard phases can provide valuable information to assess the analytical reliability of each detection method. The RR of each detection method can be assessed by dividing the difference in response between liquid and gas phase standard by the response of the liquid phase. The computed RR values can thus be used as one of the critical parameters to assess the reliability of the analytical coupling between the standard phases (liquid versus gas) and analytical method (GC versus HPLC).

### 2.2. Preparation of CC Standards in Liquid and Gas Phase

As seen in Tables 2 and 3, standards of two different phases were prepared independently for each method. The gas phase working standards (G-WS) were first prepared separately for each system by diluting gaseous primary standard (G-PS). The G-PS containing the 5 target carbonyls was purchased from Ri Gas Co., (Daejeon, Republic of Korea) containing AA (99.6 ppm), PA (20.1 ppm), BA (18.6 ppm), IV (19.6 ppm), and VA (15.1 ppm). The G-WS for the HPLC calibration was prepared at 5 concentration levels (Table 2(a)), while that for the GC at 3 concentration levels (Table 3(a)). For HPLC analysis, the G-WS of formaldehyde (FA) was also prepared separately by vaporizing formalin solution. FA was then mixed with the G-WS of 5 CCs (from cylinder) to use standard mixture of 6 CCs (Table 2(a)). In contrast, in the case of GC-based analysis, benzene (B) was instead added into the G-WS of 5 CCs as a reference compound due to its stability and good recovery. As the GC-MS conditions were not feasible to quantitate the low molecular weight compound like FA, GC-based analysis was only confined to five CCs from the cylinder (Table 3(a)). Hence, the selection of the target components by the two systems is distinguished in that FA and benzene are measured in addition to the five main CC targets for the HPLC and GC-MS, respectively.

The liquid phase WS (L-WS) for each system was also prepared independently for the comparative calibration by each system. As shown in Table 2(b), the L-WS for the HPLC-based analysis was prepared at 5 different concentration levels using the standard commercially available carbonyl-dinitrophenylhydrazine (DNPH) mix (Supelco, USA). In contrast, the L-WS for the GC-based analysis was prepared gravimetrically as shown in Table 3(b). These L-WS were made to cover three different concentration levels using the primary grade chemicals purchased at the purity of ≥99% (Sigma-Aldrich, USA). They were prepared independently from those of HPLC to avoid interfering effects of DNPH in the detection stage of GC-MS. (Note that the L-WS for HPLC analysis is made on the basis of DNPH derivatization). In the case of the GC-based analysis, the L-WS was also made to contain benzene as a reference compound along with the five target CCs for direct comparison with gas-phase standard.

### 2.3. Carbonyl Analysis by HPLC Method

To assess the relative recovery of HPLC-based calibration data between the two standard phases, the calibration results were derived from both G-WS and L-WS in a comparable manner. In the case of G-WS, each of all five standard samples (five concentrations, Table 3(a)) was pulled into the cartridge to induce derivatization with DNPH. The collection of CCs from G-WS was made by the cartridges prepacked with chromatographic grade silica (60–100 mesh) and coated with 2,4-DNPH (1 mg/cartridge) (Supelco Inc., PA, USA). All standards were collected into the cartridges at a fixed flow rate of 1 L min^−1^ for 8 minutes and regulated by a vacuum pump with an adjustable flow controller (MP-Σ 300, SIBATA, Tokyo, Japan). Teflon tubing was used to connect the Tedlar bag, DNPH cartridge, and flow controller. After each sampling, the cartridges were capped and wrapped in pouches (Supelco Inc., PA, USA). The pouches were stored in desiccators until the carbonyl analysis was performed (e.g., within 2 hours).

The carbonyl-hydrazones were analyzed by HPLC (Lab Alliance 500) equipped with a UV detector and dsCHROM software for peak integration. To initiate the HPLC-based analysis of G-WS, the cartridges were eluted slowly with acetonitrile into a 5 mL capacity borosilicate glass volumetric flask. The eluate was injected into the HPLC system equipped with a 20 *μ*L sample loop. Different carbonyl-hydrazones were separated on a Hichrom 250 × 4.6 mm ODS (octadecylsilane), 5 *μ*m reverse phase C_18_ column using a mobile phase of acetonitrile + water (6.5 : 3.5 by volume) at a flow rate of 1.5 mL min^−1^. The final calibration of the gaseous CCs was performed by injecting 20 *μ*L of each eluate taken from five different G-WS. In case of L-WS, samples of DNPH mix standard prepared at five concentration levels (Table 2(b)) were analyzed by injecting 20 *μ*L of each into the HPLC.

### 2.4. Carbonyl Analysis by TD/GC/MS Method

To perform the comparison between different analytical approaches in CC analysis, the performance of the TD/GC/MS method was also investigated between the gas- and liquid-phase standards (Table 3). To conduct the GC-based calibration, standards prepared in both phases were treated in an identical manner; each of them was initially loaded on the sorbent tube and subject to thermal desorption in a consistent manner. The sorbent tube was packed with 300 mg of the Carbopack X sorbent (mesh 60/80). Once the sorbent tube was loaded with WS, it was subjected to the adsorption/desorption cycle inside the TD system. The cold (or cryofocusing) trap in the TD system was packed with two sorbent materials of Tenax TA and Carbopack B at equivolume ratio. The selection of sorbents used in the present work was based on experiments done in our laboratory [28, 29]. The adsorption and desorption of the analytes in the TD system were carried out at 5°C (5 min) and 320°C (20 min), respectively. The carbonyl compounds were then separated on a CP-Wax column (diameter: 0.25 mm, length: 60 m, and thickness: 0.25 *μ*m) with a split ratio of 1 : 5. The column temperature was ramped at 10°C min^−1^ from an initial temperature of 40°C to the final temperature of 180°C. Helium (>99.999%) was used as a carrier gas at a flow rate of 1 mL min^−1^. The detection of carbonyl compounds was made by an MS interfaced to the GC, 70 eV EI ionization, and 200°C ion source temperature. Total ion chromatograms (TIC) mode was used over a mass range of m/z 35 to 250.

In the case of G-WS, the inlet of the sorbent tube was connected to a standard storage container (polyester aluminum: PEA) filled with gas-phase WS via Teflon tubing, and the outlet of the sorbent tube was then connected to a vacuum pump (MP-Σ 30, Shibata, Japan). The transfer of G-WS into the sorbent tube was initiated at a fixed flow rate of 100 mL min^−1^ for 1 min. The sorbent tube loaded with G-WS was then thermally desorbed to derive calibration curves for each target compound.

The same sorbent tube was also used to calibrate the L-WS. The inlet and outlet of the sorbent tube were connected with a PEA container filled with ultra-pure nitrogen and the vacuum pump, respectively. 1 *μ*L of L-WS prepared at three concentration levels was spiked on the inlet of the sorbent tube using a 10 *μ*L liquid syringe. A purge of ultra-pure nitrogen was applied to the ST prior to TD analysis to reduce the solvent effect of liquid standard at a flow rate of 200 mL min^−1^ for 5 min [30]. The purge procedure can suppress or decrease solvent (methanol) interference with target components contained in L-WS. It can eventually help minimize any possible bias in the sample adsorption stage.

## 3. Results and Discussion

### 3.1. Comparison of the CC Calibration Trends between the Two Methods

**Figure 1:**
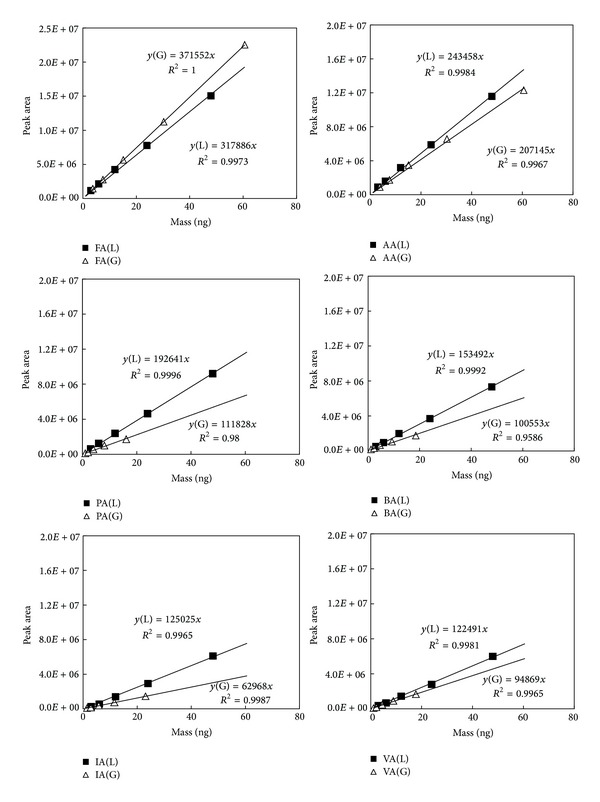
Comparison of HPLC-based calibration curves of CCs between liquid- (L) and gas-phase (G) standards.

The basic properties of each detection method used for the quantitation of CCs can be assessed by comparing the relative calibration patterns of each method. The raw calibration data for the HPLC and GC method are presented in Tables 2 and 3, respectively. As shown in Table 2, the HPLC-based analysis was tested for five odorous CCs plus FA across five calibration points. In comparison, the GC/MS-based analysis for five CCs and benzene was made as three point calibrations for both liquid and gas phase standards (Table 3). Because of the excellent linearity with high *R* ^2^ values, all GC-MS analyses were confined to three point calibration for the sake of simplicity. The results of the HPLC calibration experiments derived by both standard phases are plotted in Figure 1. Likewise, the comparable data sets derived by GC/MS are also depicted in Figure  1S.

As shown in Table 4, the results of these calibration experiments can be summarized in terms of response factor (RF) and coefficient of determination (*R* ^2^). These results are also presented to allow comparison between the two different experimental methods and between the two standard phases. The results of our experiments show that the calibration data obtained by the HPLC generally maintained higher *R* ^2^ values for all CCs, although results of PA and BA were slightly lower with 0.98 and 0.96, respectively. Likewise, the GC-based experiments also yielded fairly good calibration results with an exception of AA prepared in gas phase. In our previous study, the TD-GC analysis of the lighter CCs like AA was found to suffer from limited linearity depending on the sorbent material type used in the cryofocusing stage [28, 29].

Although the calibration results derived by both systems cannot be compared in absolute terms, their relative patterns between different compounds can be assessed very meaningfully. According to this compilation, the relative ordering in RF values of the HPLC method decreases monotonically (as 1/MW: L-WS *R* ^2^ = 0.986 and G-WS *R* ^2^ = 0.967) with increasing molecular weight (MW), regardless of standard phase. As expected, the 360 nm UV molar extinction coefficients are very similar across all analyzed carbonyl-dinitrophenylhydrazine derivatives [31]. The corresponding molar MDL's for HPLC/UV analysis of the L-WS aldehyde/ketone-DNPH mix are ~0.80 ± 0.02 pMol (see Table 5). The present work's MDL results are inconsistent with the almost constant MDL (0.04–0.06 mg L^−1^) reported for a 25 *μ*L L-WS HPLC injection of a CC-hydrazone mix [32]. The VA/FA molecular weight ratio is 86/30 = ~3. We make no attempt to reconcile our MDL results with the [32] data. The HPLC-MS MDL for PFPH derivatives (of FA, AA, PA, BA, and VA in gas samples) ranging from 0.21 ppb (FA) to 0.10 ppb (VA) [17] may generally reflect EI ionization efficiencies. More specifically, the maximum and minimum sensitivities are observed by FA and VA, respectively. As such, it is apparent that HPLC system is favorable to maintain enhanced sensitivity for the lighter CCs relative to the heavier CCs (on a per unit-weight basis—a molar basis, it is essentially constant). The cause of such systematic differences in HPLC-based analysis has been explored in our recent study [20].

In contrast, an opposing trend is apparent with the results derived by the GC method. The GC-based calibration data consistently indicate that the magnitude of RF values increases fairly systematically with increasing MWs, although their sensitivities are generally lower than the reference compound, benzene. (*N.B*.: the 70 eV EI ionization cross sections increase linearly with MW.) As a result, the RF's (and also MDL's in mass units) will be essentially constant, irrespective of MW assuming quantitative recovery. Hence, the RF's (*i.e*., slope of peak area *versus* analyte weight) behavior for the GC-MS and HPLC-UV methods observed in this study are readily explainable in terms of EI ionization cross sections (see below for explanation, Section 3.3, last paragraph) and molar extinction coefficients, respectively.

### 3.2. Basic Quality Assurance of HPLC and GC-MS Method

The results of our comparative study show that the performance of the two systems is highly contrasting in many respects. The observed contradictory trend in relative sensitivity between the two methods is in fact reflected further, if comparison is extended in terms of the basic quality assurance (QA) parameters like precision or detection limits. To obtain the analytical precision for the CC determinations, the relative standard error of the mean (RSE: %) was assessed based on the triplicate analyses of each standard phase by each method (Table 5). Comparison of RSE values of each method suggests that distinctions in reproducibility can be made between standard phases rather than intercompound relationships.

To conduct CC measurements, one needs to initiate the sampling step in which analytes in ambient air are enriched on an appropriate sampling medium via sorption [33, 34], cryotrapping [33, 35], or derivatization [24, 36, 37]. The detectability of CC is then determined not simply by the sensitivity of a given instrument but by its interactive relationship with those sampling methodologies. The detectability of each compound was also assessed in terms of method detection limit (MDL). The MDL for each CC was calculated by referring to the guidelines in 40 CFR Part 136, Appendix  B [38] as the product of the standard deviation of seven replicates and the Student's *t*-value at the 99% confidence level (*t* = 3.14 at 6 df).

The present work's detection limits for both systems are shown in Table 5. Evaluation of DL values shows an interesting trend. For both methods, detectability is greatly distinguished so that the maximum sensitivity is attained by lighter (HPLC) and heavier CCs (GC) in full compliance with the relative properties discussed above. These observed trends are due in part to molar extinction coefficients and EI ionization cross-sections considerations, thus reflecting the physics of the detectors being used. Explanations for the observed difference between the two systems can be sought mainly from the coupling effect between sample treatment procedures and the instrumental detection system such as effective derivatization of lighter CCs for HPLC analysis and preference of heavier CCs for TD-GC analysis.

The HPLC method is generally based on the derivatization technique to form a stable product in the reaction between analytes and the derivatizing reagent (for example: 2,4-dinitrophenylhydrazine [39]). In contrast, the GC-based analysis can be simplified, if coupled with the thermal desorption (TD) technique, while the adoption of the initial reaction (e.g., with derivatization) can be a selectable option [21]. A 1992 review reports that solid sorbent sampling with Tenax followed by TD/GC-FID analysis yielded CC detection limits of <0.1 ppb for 3 L samples [23]. Later in 2008, detection limits in the range of 0.75 to 2.33 ng (~0.1 ppb calculated for 3 L samples) were reported for AA, PA, BA, IA, and VA using cold trapping/TD/GC-FID analysis; calibration properties were observed to be moderately dependent on TD settings [33]. A potentially attractive method that could be used for near real time general VOC analysis at the pptv level is proton transfer reaction mass spectrometry (PTR-MS) [40]. For example, a detection limit of 78 pptv for FA has been reported using a PTR-MS equipped with a −40°C sampling cryo-dehumidifier (containing amorphous silicon), 250 sccm air sampling rate, and 5 s data acquisition time on m/z 31 (sample volume consumed ~21 mL) [41].

### 3.3. Relative Recovery (RR) of CC Standards between Different Methods

To analyze CC in gaseous media by the GC or HPLC methods, the use of preconcentration technique [18] is often a requisite choice to handle large sample volumes to overcome the trace analyte abundances per given sample matrix. Thus, the reliability of each method is determined by the cumulative effects of experimental biases stemming from the initial sample collection stage to the final detection/data analysis stages. Considering that the quantitation of CCs in gaseous media (air) is commonly made against standard prepared in liquid phase, relative recovery (RR) between standard phases can be used as a critical variable to assess the reliability of each method. Hence, the experimental performance of the two major detection techniques for CCs can be evaluated based on direct evaluation of the RR between liquid and gas phase standards.

It is interesting to note that HPLC system exhibits generally enhanced reproducibility from liquid phase standards to reflect the relatively simple steps involved in the calibration of L-WS. In contrast, enhanced reproducibility of gaseous CC standard is also apparent in the GC analysis. To learn more about the possible bias stemming from the use of different phase standards, the difference in RF values for a given method can be assessed in terms of RR between liquid and gas phases. Hence, as a means to assess the RR for a given compound, the percent difference (PD) was computed by dividing the observed RF differences between the two standard phases with that of liquid phase (Table 4).

The resulting PD values derived for each method also seem to comply with the patterns seen from the relative sensitivity derived by the interactive relationship between standard phases and instrumental setups. In the case of HPLC, the PD values tend to increase with the increasing MWs of the CCs. As such, comparison of relative ordering in RF values and of PD values consistently suggests that the use of HPLC should be less reliable for the heavier CCs than the lighter ones. On the other hand, an opposing trend is evident from the GC-based calibration data, as the PD values tend to decrease with increasing MWs. As such, the overall results of our comparative analysis confirm a strong and consistent trend in the analysis of CC analysis for a given instrumental system.

Table 4 presents the molar RF's (MRF) [defined as RF∗MW/1,000,000] obtained using HPLC-UV analysis. For the L-WS, all analyzed CCs have similar MRF values ranging from 9.6 (FA, *e* = 17.5 × 10^3^) to 11.2 (PA, *e* = 20.1 × 10^3^) and the MRF correlates reasonably well with the literature molar extinction coefficient (*e*) for the carbonyl-DNPH derivatives. On the other hand, the MRF values for the G-WS showed a general decrease with increasing MW ranging from 11.1 (FA, MW = 30) to 5.4 (IA, MW = 86) excluding VA (MRF = 8.2, MW = 86). Also shown in Table 4 are the relative recovery factors (RRF) with respect to benzene set at 100% for TD-GC-EI-MS analysis of L-WS and G-WS. Based on work done in our laboratory, the aromatics (*e.g*., benzene) can be safely assumed to have near quantitative RFs. The relative EI ionization cross sections  (*s*_*r*_) with respect to N_2_ (*s*_*r*_ = 1.00) can be estimated from molecular polarizability (a) as follows: for hydrocarbons *s*_*r*_ = 0.50*a* − 0.05 and (b) for VOCs (other than hydrocarbons) *s*_*r*_ = 0.36*a* + 0.30 [42]. The molecular polarizabilities (a) were calculated using an additivity scheme based on atomic polarizabilities [43]. The following *s*_*r*_ values (in parentheses) were estimated for B (5.15), AA (1.92), PA (2.58), BA (3.24), and IA/VA (3.90).

From the ionization response factors (=*s*_*r*_/MW), the RRFs in parentheses relative to B (=100%) were estimated for AA (66%), PA (67%), BA (68%), and IA/VA (69%). Hence, very evidently, the RRF values for AA by GC-MS are very poor at 2.4% and 0.56% for L-WS and G-WS, respectively. The GCMS RRFs do however improve significantly in going from AA to VA/IA, approaching the values estimated from *s*_*r*_ considerations. For example for IA, the RRF values were 61.3% and 60.4% for L-WS and G-WS, respectively. These values are close to the theoretical value of 69%, suggesting an RF of IA to be 88%. For the interested reader, the reported RRs ranged from 14% to 213% for 31 different CCs (C_1_–C_13_) using DNPH cartridge-RRLC-UV analysis using a MeOH/THF/*i*PrOH/H_2_O HPLC mobile phase [44].

## 4. Conclusion

In this research, the basic characteristics of the two key experimental approaches available for carbonyl analysis, namely GC and HPLC were investigated against both liquid and gas phase standards. The experimental uncertainties in the quantitative analysis of CCs were then assessed by examining the compatibility of calibration results derivable by all four combinations between two experimental methods and two standard phases. Considering that the availability of gas phase standard is virtually not possible for all different volatile components in air, information concerning compatibility between different standard phases can be a critical component in validating the feasibility of liquid phase standard in the quantitative analysis of gas samples. Moreover, the reliability of a given instrumental method needs to be assessed thoroughly for the target compounds, if multiple instrumental methods are available for the analysis.

The overall results of our study confirm a strong consistency in the analytical properties of the GC and HPLC methods in the quantitation of CCs in air. If the feasibility of a given method is assessed in terms of relative recovery, the patterns contrast greatly between the two methods. Firstly, the HPLC method can yield the most reliable results for the lighter CCs like FA and AA; in contrast, the GC method is found to yield enhanced recoveries in the higher CCs like BA, IA, and VA. Although the calibration of PA can be made with the high coefficient of determination from all different coupled systems, it can suffer most significantly from the recovery. The overall results of our study thus confirm that the maximum reliability of CC analysis can be attained, if their analysis is made for a given species with the optimum coupling between the instrumental method and standard phases. Considering that there are limitations in the applicability of each method in the detection of CCs, one needs to put more effort to extend their applicability (e.g., the use of improved derivatization techniques, e.g., PFPH) to yield the data sets for the maximum number of CCs with the least bias.

## Supplementary Material

The operational conditions of the instrumental system and calibration results of gas chromatography are provided in the supplementary material (SM). The detailed information for the analysis of carbonyl compounds (CC) are shown in Table S1. In addition, Fig S1 depicts GC-based calibration results of carbonyl compounds (CC) between liquid- (L) and gas-phase (G) standards.

## Figures and Tables

**Table 1:** Basic information of the target carbonyl compounds (CC) selected in this study.

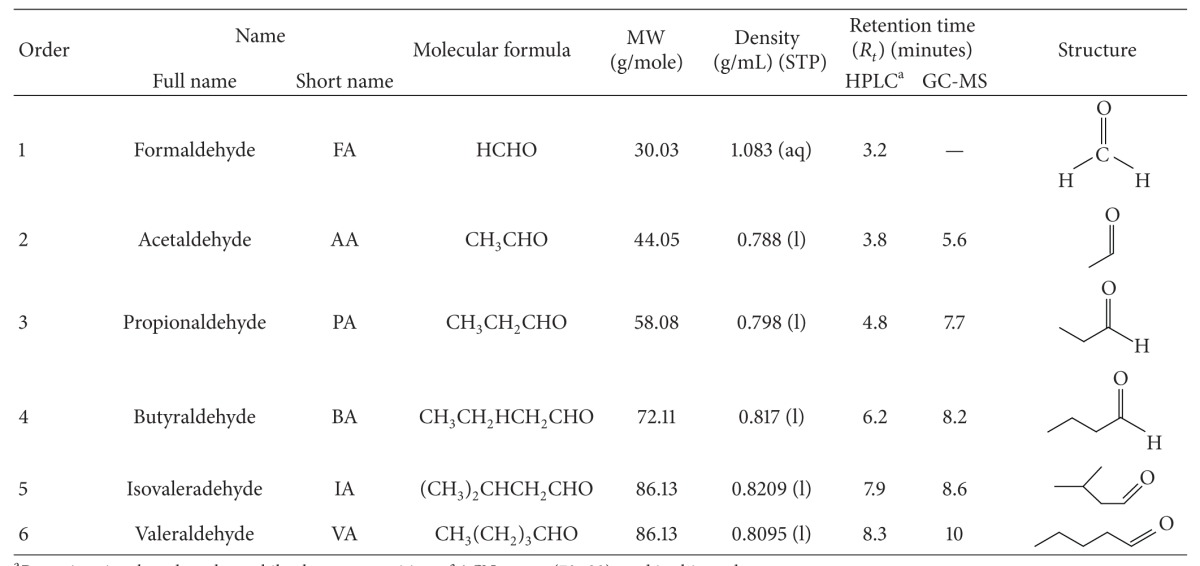

^a^Retention time based on the mobile phase composition of ACN : water (70 : 30) used in this study.

**Table:** (a) Calibration of gas-phase CC standard by HPLC/DNPH cartridge method

Order	FA	AA	PA	BA	IA	VA
Concentration of CC standard (ppb) for 5-point analysis						
1	96.1	65.1	13.1	12.2	12.8	9.90
2	194	132	26.6	24.6	25.9	20.0
3	387	264	53.0	49.2	51.9	40.0
4	773	527	106	98.0	104	80.0
5	1,559	1,058	213	198	208	160

Calculated mass (ng) of CC injected into HPLC^a^ assuming no losses in derivatization and extraction						
1	3.79	3.79	1.01	1.16	1.46	1.12
2	7.58	7.58	2.02	2.31	2.91	2.24
3	15.2	15.2	4.03	4.63	5.83	4.49
4	30.3	30.3	8.06	9.26	11.7	8.98
5	60.6	60.6	16.1	18.5	23.3	18.0

Peak area						
1	1,430,395	882,104	114,854	164,484	74,796	80,079
2	2,754,983	1,733,480	222,048	297,748	146,260	177,013
3	5,650,140	3,472,465	577,168	640,128	375,504	449,499
4	11,240,906	6,552,152	1,007,331	1,059,943	728,865	894,010
5	22,526,453	12,305,560	1,718,513	1,742,954	1,454,395	1,660,223

^a^For each calibration point, 8 L of gaseous CC standard is sampled by the cartridge and these CCs are extracted by 5 mL acetonitrile. As 20 *μ*L of extract is injected into HPLC, the actual mass (ng) of CC loaded onto HPLC is computed as the total quantity of each CC contained in 20 *μ*L extract.

**Table:** (b) HPLC calibration results for liquid-phase CC standard

Concentration	Loading	Peak area					
(ng *μ*L^−1^)	mass (ng)^a^	FA	AA	PA	BA	IA	VA
0.15	3	1,150,435	886,294	606,404	502,987	259,055	358,175
0.30	6	2,137,986	1,584,941	1,247,462	940,570	555,295	666,852
0.60	12	4,243,306	3,183,149	2,388,591	1,987,032	1,391,763	1,425,906
1.20	24	7,763,079	5,875,879	4,646,142	3,691,885	2,922,397	2,786,051
2.40	48	15,043,330	11,578,866	9,202,951	7,322,176	6,099,023	5,976,525

^a^Aldehyde/ketone-DNPH mix (Supelco): liquid phase standard is prepared to have equal mass for all target compounds per unit volume.

**Table:** (a) GC-based calibration of gas-phase CC standard

Order	B^a^	AA	PA	BA	IA	VA
Concentration (ppb)						
1	40.0	199	40.2	37.2	39.2	30.2
2	80.0	398	80.4	74.4	78.4	60.4
3	160	797	161	149	157	121

The actual mass (ng) of CC injected into TD/GC^b^						
1	12.8	35.9	9.5	11.0	13.8	10.6
2	25.6	71.8	19.1	21.9	27.6	21.3
3	51.1	144	38.2	43.9	55.2	42.6

Peak area						
1	1,835,850	66,043	114,313	615,231	1,105,826	808,071
2	3,516,064	101,214	218,491	1,198,036	2,257,147	1,578,254
3	6,794,287	103,975	431,169	2,428,141	4,484,753	3,306,639

^a^Because of limitation in the analysis of FA, benzene (B) was analyzed in place of FA.

^b^Flow rate = 100 mL min^−1^, loading time = 1 min, and loading volume = 100 mL.

**Table:** (b) GC-based calibration of liquid-phase CC standard

Order	B	AA	PA	BA	IA	VA
Concentration (ng *μ*L^−1^) of CC injected into GC^c^						
1	10.1	27.2	9.03	9.30	9.02	9.17
2	20.2	54.4	18.1	18.6	18.0	18.3
3	40.4	109	36.1	37.2	36.1	36.7

Peak area						
1	1,502,193	76,511	36,105	425,976	641,460	612,205
2	2,784,977	172,068	113,520	954,543	1,503,596	1,489,447
3	5,361,966	351,382	234,837	1,957,700	2,992,220	3,077,189

^c^GC injection volume: 1 *μ*L.

**Table 4:** Comparison of relative recovery of each CC between different standard phases for a given method.

Method	Type	FA/B	AA	PA	BA	IA	VA
HPLC-UV	RF(L)	317,886	243,458	192,641	153,492	125,025	122,491
	RF(L) ∗ MW^a^	9.6	10.7	11.2	11.0	10.8	10.5
	*R* ^2^(L)	0.997	0.998	1.000	0.999	0.997	0.998
	RF(G)	371,552	207,145	111,828	100,553	62,964	94,871
	RF(G) ∗ MW^a^	11.1	9.1	6.3	7.3	5.4	8.2
	*R* ^2^(G)	1.000	0.997	0.980	0.959	0.999	0.997
	PD	−17	15	42	34	50	23

GC-EI-MS^b^	RRF(*σ*_*r*_)^c^	100	66	67	68	69	69
	RF(L)	134,475	3,197	6,341	52,065	82,455	82,598
	Rel RF(L)	100	2.4	4.7	39	61	61
	*R* ^2^(L)	0.9961	0.9968	0.976	0.9967	0.9961	0.9923
	RF(G)	134,336	747	10,874	55,232	81,249	76,956
	*R* ^2^(G)	0.9979	0.906	0.9953	0.9998	0.9999	0.9986
	Rel RF(G)	100	0.56	8.1	41	60	57
	PD	0.10	76.65	−71.49	−6.08	1.46	6.83

^a^Divided by 1,000,0000 to give small numbers for convenience—essentially molar RF figure of merit; ^b^because of limitation in the analysis of FA, benzene (B) is analyzed in replacement of FA, and ^c^RRF(*σ*_*r*_) is based on estimated EI total ionization cross sections (see text for details).

**Table:** (a) Precision (relative standard error (RSE): in %)

		FA/B	AA	PA	BA	IA	VA
HPLC	Gas^a^	1.81 (FA)	1.97	2.33	2.26	1.76	1.92
	Liquid^b^	0.46 (FA)	0.80	0.92	0.67	0.70	0.78

GC-MS	Gas^c^	0.23 (B)	0.24	0.35	0.55	0.03	0.49
	Liquid^d^	3.46 (B)	1.14	4.32	2.61	3.14	3.34

^a^Loading volume = 8 L (8 min at a flow rate = 1 L min^−1^) of 121 (VA) to 1171 ppb standard (FA); ^b^injection amount = 20 *μ*L of 0.6 ng *μ*L^−1^ (12 ng); ^c^loading volume = 100 mL (1 min at flow rate = 100 mL min^−1^) of 38 (VA) to 250 ppb standard (AA); and ^d^1 *μ*L injection of 18 (VA) to 54 ng *μ*L^−1^ (AA) liquid standard.

**Table:** (b) Detection limits

		Units^a^	FA/B	AA	PA	BA	IA	VA
HPLC	Gas^b^	pg	21.0 (FA)	37.7	69.8	77.6	123.9	82.2
		ppb	0.53 (FA)	0.65	0.92	0.82	1.10	0.73
	Liquid^c^	pg	27.6 (FA)	36.0	45.5	57.2	70.2	71.6
		ppb	0.70 (FA)	0.63	0.60	0.61	0.62	0.64
		pMol	0.92 (FA)	0.82	0.78	0.79	0.83	0.82

GC-MS	Gas^d^	ng	0.07 (B)	9.68	0.81	0.16	0.11	0.11
		ppb	0.02 (B)	5.37	0.34	0.05	0.03	0.03
	Liquid^e^	ng	0.07 (B)	2.97	1.50	0.18	0.12	0.12
		ppb	0.02 (B)	1.65	0.63	0.06	0.03	0.03

^a^To calculate concentration in ppb, the total sample volumes are assumed as 8 (HPLC) and 1 L (GC-MS); ^b^loading volume = 8 L (8 min at a flow rate = 1 L min^−1^) of 2.7 (VA) to 26 ppb standard (FA); ^c^injection amount = 0.5 ng (20 *μ*L of 0.025 ng *μ*L^−1^); ^d^loading volume = 50 mL (1 min at flow rate = 50 mL min^−1^) of 0.76 (VA) to 4.98 ppb standard (AA); and ^e^1 *μ*L injection of 0.29 (IA) to 0.87 ng *μ*L^−1^ (AA) liquid standard.
