# Supplementary material for: An Exploration on the Suitability of Airborne Carbonyl Compounds Analysis in relation to Differences in Instrumentation (GC-MS versus HPLC-UV) and Standard Phases (Gas versus Liquid)
Source: ScientificWorldJournal. 2014 Feb 25;2014:308405. doi: 10.1155/2014/308405 (PMC3956549; doi:10.1155/2014/308405)
Supplement: Supplementary file 1 — The operational conditions of the instrumental system and calibration results of gas chromatography are provided in the supplementary material (SM). The detailed information for the analysis of carbonyl compounds (CC) are shown in Table S1. In addition, Fig S1 depicts GC-based calibration results of carbonyl compounds (CC) between liquid- (L) and gas-phase (G) standards. [file 308405.f1.docx]

**Date: 17 Feb. 2014 (V5)**

**Supplementary Information**

**An exploration on the suitability of airborne carbonyl compounds analysis in relation to differences in instrumentation (GC vs. HPLC) and standard phases (gas vs. liquid)**

Ki-Hyun Kim, Jan E. Szulejko, Yong-Hyun Kim, Min-Hee Lee

Department of Civil and Environmental Engineering, Hanyang University,

222 Wangsimni-Ro, Seoul 133-791, Korea

Corresponding author:

E-mail: [kkim61@nate.com](mailto:kkim61@nate.com)

Tel: 82-70-756-9151

Previous affiliation:

Dept. of Environment & Energy, Sejong University, Seoul, Korea 143-747

Table 1S. The operational conditions of the instrumental system for the analysis of carbonyl compounds^a^

A.HPLC/UV system

| 1) Injector |  |  |  |  | 3) UV detector |  |  |
| --- | --- | --- | --- | --- | --- | --- | --- |
| Sample injection | 20 |  | µL |  | Model 500, Lab Alliance (USA) | | |
| volume |  |  |  |  | Wavelength | 360 | nm |
| 2) Pump |  |  |  |  | 4) Column |  |  |
| Flow rate | 1.5 |  | mL·min^-1^ |  | Model | 5 C 18, Hichrom (UK) | |
| Mobile phase | ACN : H_2_O | = | 7 : 3 |  | Particle size | 5 | µm |
| Analysis time | 15 |  | min |  | Temp | 20 | ℃ |

a] HPLC (Series 1500, Lad Alliance, USA)

B. TD/GC/MS system^b^

| **GC (SHIMADZU GC-2010, JAPAN), MS (SHIMADZU GCMS-QP2010, JAPAN)** | | | | | |
| --- | --- | --- | --- | --- | --- |
| Column: CP Wax (diameter: 0.25 mm, length: 60 m, and film thickness: 0.25 µm) | | | | | |
| Oven setting | |  | Detector setting | | |
| Oven temp: | 40 ℃ (5 min) |  | Ionization mode: | | EI (70 eV) |
| Oven rate: | 10 ℃ min^-1^ |  | Ion source temp.: | | 200 ℃ |
| Max oven temp: | 180℃ (1 min) |  | Interface temp.: | | 200 ℃ |
| Total time: | 20 min |  | TIC scan range: | | m/z 35~250 |
| Carrier gas:  Carrier flow: | He (99.999%)  1 mL min^-1^ |  | Threshold: |  | 100 |
| **Thermal desorber (Unity, Markes International, UK)** | | | | | |
| Cold trap: | Carbopack B+ Tenax TA | | |  |  |
| Split ratio: | 1:5 |  | Trap low: |  | 5 ℃ |
| Split flow: | 5 mL min^-1^ |  | Trap high: |  | 320 ℃ |
| Trap hold time: | 10 min |  | Flow path temp: | | 150 ℃ |
| **Sampling Tube** | | | | | |
| Absorbent: | Carbopack X (300 mg) | | |  |  |
| Desorb time: | 20 min |  | Temp.: |  | 320 ℃ |

^b^ TD (UNITY, Markes International, Ltd., UK)

Fig 1S. Comparison of GC-based calibration curves for carbonyl compounds (CCs) between liquid- (L) and gas-phase (G) standards.
